# Validity and Usability of Physical Activity Monitoring in Patients with Chronic Obstructive Pulmonary Disease (COPD)

**DOI:** 10.1371/journal.pone.0157229

**Published:** 2016-06-15

**Authors:** Tobias Boeselt, Marc Spielmanns, Christoph Nell, Jan Hendrik Storre, Wolfram Windisch, Lena Magerhans, Bjoern Beutel, Klaus Kenn, Timm Greulich, Peter Alter, Claus Vogelmeier, Andreas Rembert Koczulla

**Affiliations:** 1 Department of Medicine, Pulmonary and Critical Care Medicine, University Marburg, Marburg, Germany; 2 Department of Pneumology, University Hospital, Freiburg, Germany; 3 Department of Pneumology, Cologne Merheim Hospital, Cologne, Germany; 4 Faculty of Health, Department of Pneumology, University of Witten/Herdecke, Germany; 5 Medical Clinic and Pulmonary Rehabilitation in Leverkusen (April), Remigius Hospital, Leverkusen Opladen, Germany; 6 Schoen Klinik Berchtesgadener Land, Schönau am Königssee, Germany; Research Center Borstel, GERMANY

## Abstract

**Background:**

A large proportion of COPD patients do not achieve the recommended level of physical activity. It is suggested that feedback on the level of activity by using an activity monitoring device (PAM) increases awareness and may stimulate patients to increase their physical activity in daily life. Our objective was to assess the validity and usability of a simple and low-cost physical activity monitor (Polar A300^™^) when compared with the validated and established Bodymedia-SenseWear^™^ (SWA) device.

**Methods:**

To assess the diagnostic equivalent, two different PAM devices were used in parallel in 20 COPD patients GOLD I to IV during 3 consecutive days of daily life. Both systems were compared in terms of steps, calories burned, daily activity time and metabolic equivalents using linear regression analysis and Bland-Altman plots. Practical usability was examined by a 16-item-questionnaire.

**Results:**

High correlations of both devices were observed with regard to the sensed step count (r = 0.96; p < 0.01) and calories burned (r = 0.74; p < 0.01), and a lower correlation of daily activity (r = 0.25; p < 0.01) was found. Data analysis over 3 days showed that 90% of the steps (95% CI -4223 to 1887), 100% of the calories (95% CI -2798 to 1887), 90% of the daily activity data (95% CI -12.32, 4065) and 95% of the MET (95% CI -3.11 to 2.75) were within the limits of agreement. A favorable usability (system-, information- and interface quality) of the A300^™^ device was shown (p < 0.01).

**Conclusion:**

The A300^™^ device with easy practical usability was shown not to be inferior for assessment of physical activity time, step count and calorie consumption in COPD patients when compared with the SWA. It is suggested to consider widespread available devices as commonly used for monitoring recreational sporting activities also in patients for assessment of physical activity in daily life.

## Introduction

In patients with COPD from mild to very severe stages, physically active is considered of great importance for adequate disease management [1–3]. Compared with healthy controls, patients with COPD have significantly reduced duration, intensity and step counts (number of movements per day) of physical activity [4–6]. The daily activity decreases from COPD degree I to COPD IV [7]. On average, the COPD patients achieve a final daily step count of 5584 ± 3360 [steps/d] [4]. On the other hand it has been well documented that increased physical activity in COPD patients leads to fewer hospitalizations and a reduced mortality rate [6, 8, 9]. A higher physical activity also appears to have an impact on the stiffness of the arterial vessels and may therefore reduce the risk of cardiovascular comorbidities [10]. Despite existing concrete recommendations and the importance of physical activity in patients with COPD, it seems difficult for the majority of COPD patients to meet the recommended amount of daily physical activity [11, 12]. A recent study showed positive effects on physical activity by monitoring measurements in COPD [5, 13, 14] and was well tolerated [15]. Particularly, the daily number of steps and the daily activity time was proven to be the most valid measurement parameters [16]. The regular use of a physical activity monitor (PAM) could therefore benefit COPD patients to achieve the required daily physical activity. Meanwhile, various PAMs have been evaluated for the measurement of physical activity in COPD patients [14]. Essentially, the total energy consumption (Total Energy Expenditure = TEE) and energy consumption through physical activity (Activity Energy Expenditure = AEE) were detected. In comparison with the gold standard for energy consumption measurement (Double-Labeled Water Method) the Bodymedia Sensewear^™^ (SWA) showed sufficient accuracy in the detection of TEE and AEE [17]. Consequently, the SWA has been used as a standard tool in several COPD studies [18]. However, the SWA is very expensive, not always affordable and comfortable to wear for all patients. Therefore, we studied the validity and usability of a simple and low-cost PAM (Polar A300^™^) in comparison to the well validated and established Bodymedia SenseWear^™^ in COPD patients.

## Material and Methods

### Design and Setting

This was a prospective single-center study. Trial registration number German Clinical Trial Register (DRKS): 00009778. The study was approved by the Ethics Committee of the Medical Faculty of the University of Marburg (No 111/15).

### Study Population

20 patients, aged 40 to 90 years, suffering from COPD GOLD I to IV were included into the study. All patients contacted were involved in an outpatient, multidisciplinary pulmonary rehabilitation program at the Philipps University Marburg. Participation was voluntary and previously a written consent has been obtained. Participants were subjected to a pulmonary function test (spirometry and body plethysmography, Fa. JAEGER^™^) and following an instruction and training in the correct application of the systems according to the manufacturer's recommendations (SWA and A300^™^).

### Devices

**Fig 1:**
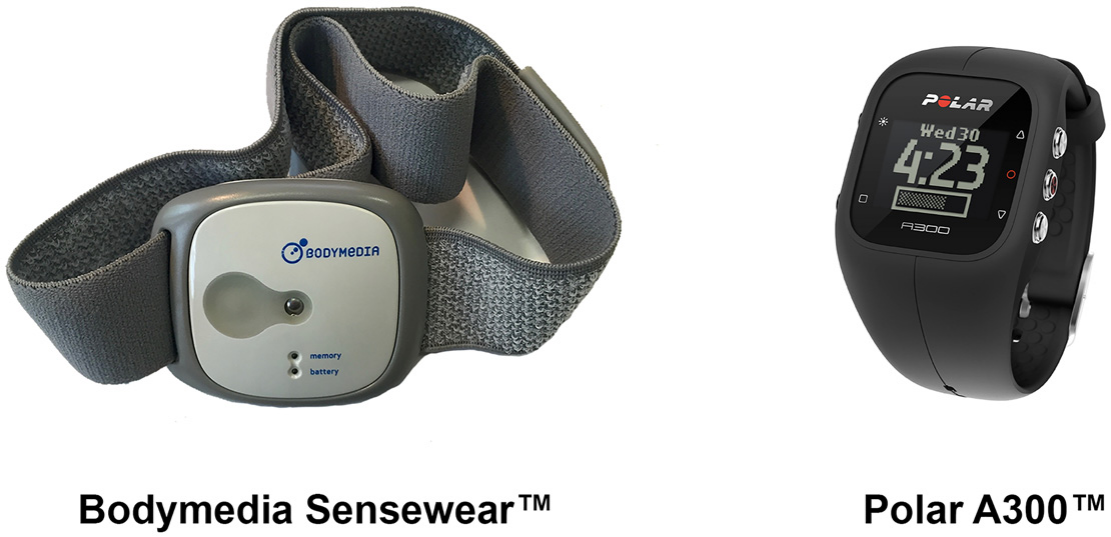
Bodymedia Sensewear^™^ and Polar A300^™^Devices.

In the present study the Polar A300^™^ device (Polar Electro Oy, Finland) was compared with multisensory accelerometer SenseWear^™^ (Body Media, Pittsburgh, PA, USA) (Fig 1). For further details, see Table 1. Both devices are working with a 3D accelerometer measuring movements in three different levels. In addition, the SWA also contained a sensor to measure the skin temperature. The data of both devices were connected via a data cable to the PC/software and stored there. The Polar Clock was worn on the left wrist and the SWA on the left arm.

**Table 1:** Details of the devices.

Device	Technology	Output
**Polar A300**^**™**^	High sensibility 3D acceleration sensor	Steps
	Data transfer via USB	Calories
	Bluetooth SMART	Daily activity
**Bodymedia Sensewear**^**™**^	High sensibility 3D acceleration sensor	Steps
	Sensors for heat flux	Calories
	galvanic skin response and temperature	Daily activity
	Data transfer via USB	Mean MET

**Fig 2:**
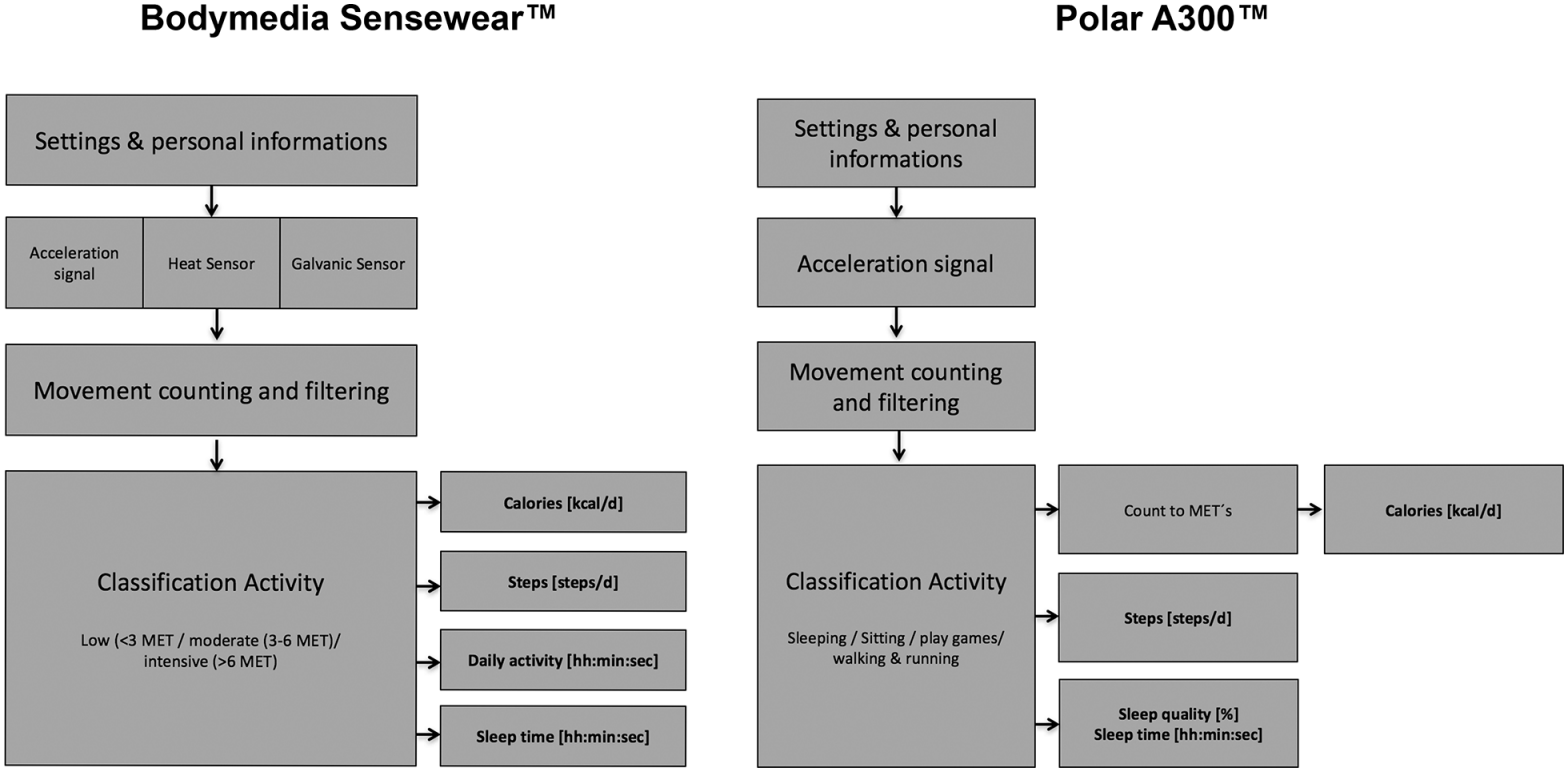
SWA and Polar method for calculating/measuring steps, calories, daily activity and MET.

Fig 2 shows the workflow of the A300^™^ to calculate the steps, calories and daily activity time based on the intensity of the movement and the calculated MET Score. At the beginning personal data (age [y], sex [f / m], height [cm], weight [kg]) were entered into the clock. The 3D-acceleration sensor registered the movements and classified them depending on their intensity in different metabolic equivalents of tasks (MET) and number of steps. For the calculation of the MET the calories were calculated. Every movement count on the daily activity time. MET is a physiological measure expressing the burned calories of physical activities. One MET is defined as 1 kcal consumption per kg of body weight and hour. The following MET levels based on the physical activity guidelines of the “Office of Disease Prevention and Health Promotion” (ODPHP). Further details are mentioned in www.health.cov.

- Light-intensity aerobic activity = 1.1 to 2.9 METs
- Moderate-intensity activity = 3.0 to 5.9 METs
- Vigorous-intensity aerobic activity = 6.0 or > METs

### Validity Study: Comparison of both devices

Patients were provided at the same time with both devices and asked to wear them for 3 days continuously (24 hours / day). They were informed about the position at which the equipment must be replaced after they have changed clothes or showered. After 3 days they gave back both of the PAMs and the data analyzed. Data collection took place under everyday conditions in which the systems were compared regarding the following parameters: steps, calories, daily activity time and quality of sleep. In a standardized, one-hour activity log, the SWA was measured in COPD patients against an indirect calorimetry and has shown a correlation of the energy consumption of r = 0.76 (95% CI 0.54–0.91). Compared to the "double labeled water" method, the SWA showed an intra—class correlation of 0.76 (95% CI 0.49–0.90) in the measurement of energy consumption in women with COPD over a period of 14 days [18].

### Usability Study

For the usability assessment we also used a study design where COPD patients, who had never used either device before, were provided at the same time with both devices for 3 consecutive days continuously (24 hours / day). The usability was evaluated with a 16-item usability questionnaire. Obtaining an overall satisfaction score is done by averaging the four sub-scales of System Quality (the average of items 1–6), Information Quality (the average of items 7–12), and Interface Quality (the average of items 13–16). Patients completed the questionnaire concerning the following items: manageability, usability and acceptability for both devices. The questionnaire included a 7 point Likert scale (1 = strongly agree to; 2 = true; 3 = applies to part; 4 = neutral, 5 = does not apply to part; 6 = not applicable; 7 = strongly disagree). The Post-Study System Usability Questionnaire (PSSUQ) is highly reliable (Cronbach's a = .94) [19].

### Inclusion and exclusion criteria

In the study, patients with COPD stage I to IV, a signed informed consent, the ability to walk and the age of 40 to 90 years were included. Patients with lack of mobility, paralysis of the arm, diseases that exclude any physical activity (e.g. congestive heart failure, acute pneumonia, recent surgeries.) were excluded.

### Primary and secondary endpoints

The combined primary endpoint was the comparison of the step recording, calories burned and activity time of the SWA (Body Media Inc., US) with the A300^™^ clock (Polar Electro Oy, Finland). As secondary endpoint the 16-item questionnaire was used assessing the usability (ease of use, usefulness, and acceptability) with a 7 points Likert scale.

### Statistical analysis

Data of the SWA and A300^™^ device included steps, calories burned and physical activity time and MET (SWA). The MET, as an expression of intensity and energy consumption, was calculated for A300^™^ by dividing calories burned per hour by the physical activity time per body weight. To determine the relationship of measured parameters of both devices Bland-Altman inclusive the limits of agreement (LoA) and confidence interval (CI) were calculated [20]. In addition, the intra-class correlation (ICC) was calculated for each parameter. Since not directly provided by the A300^™^ device, MET was calculated based on calorie consumption per hour and body weight applying the definition above. For comparison of data as assessed by both device, linear regression and Bland-Altman plots were applied in standard manner. For proof of the equality of the two devices, an equivalence test was used. For this, the 95% confidence interval (95% CI) for the mean difference was calculated and tested, whether this are in the equivalence range -δ to δ. Regarding the usability an average value was calculated for each domain in the questionnaire. Differences between SWA and A300^™^ were calculated by using the Mann-Whitney U test. For data analysis, SPSS 22 (IBM GmbH, Ehningen, Germany) were used. Statistical differences were assumed for p < 0.05. Unless specified otherwise, means +/- SD were given.

### Results

20 of the 27 screened COPD patients fulfilled the inclusion criteria and completed the entire clinical trial according to the protocol. The collected lung function parameters, as well as the anthropometric data are shown in Table 2.

**Table 2:** Anthropometric Data and parameter for lung function (n = 20).

Variable	Patients (Mean ± SD)
Age (years)	66.4 ± 7.4
Sex (m/f)	17 / 3
BMI [kg Body weight/m2 body surface area]	28.9 ± 5.4
6-MWT [m]	398 ± 144
Borg-Scale [points]	2 ± 1
FEV_1_ [liter]	1.87 ± 0.9
FEV_1_% pred	63.4 ± 25.8
Tiffeneau-Index (FEV_1_ / FVC)	50.5 ± 15.1
Gold Grade (I/II/III/IV)	7 / 8 / 2 / 3
Current Smoker/Ex-Smoker	3 / 17
Pack years	35.1 ± 11.2

M = male; f = female; BMI = Body-Mass-Index; 6-MWT = Six-Minutes-Walking test; FVC = forced vital capacity; FEV1%pred = forced expiratory volume % set point; FEV1%FVC = forced expiratory volume % to forced vital capacity; SD = standard deviation; Pack years = Number of Pack years (20 cigarettes per day over a period of one year)

Based on spirometry measures (FEV_1_% pred.) and symptoms, the GOLD guidelines classifies the patients in four different categories. GOLD I means a mild severity with a FEV_1_% pred. of ≥ 80, while the GOLD II (moderate) patients only has FEV_1_% pred. of 50–79. GOLD III (severe) patients only has a pred. FEV_1_% of 30–49 and GOLD IV (very severe) patients < 30 FEV_1_% pred. or chronic respiratory failure. Further details are mentioned in www.goldcopd.org.

### Validity data: Comparison of Steps, Calories, activity time, and Mets among both devices

**Fig 3:**
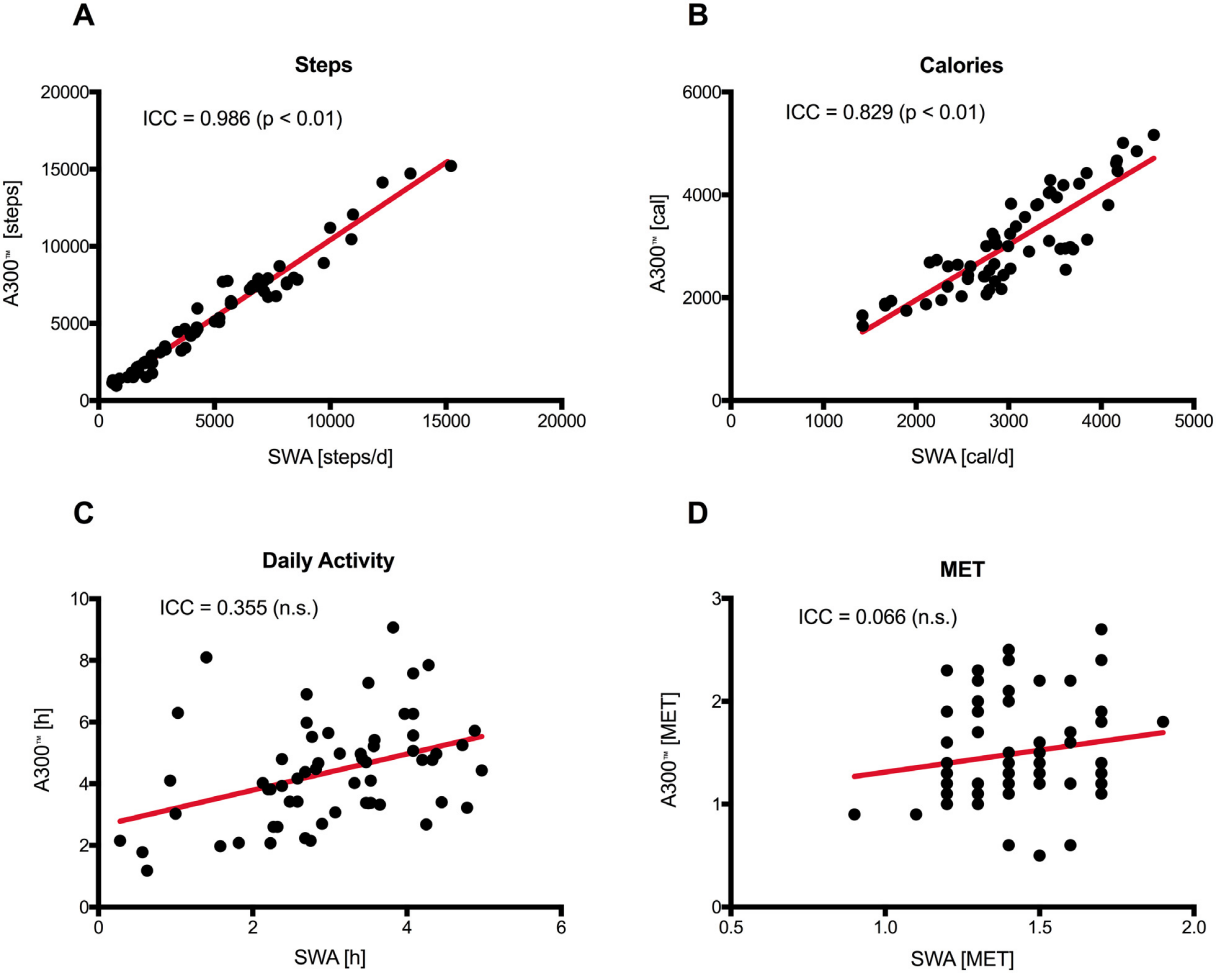
Regression analysis and Identity plots. Steps (A), calories (B), daily activity (C) and MET (D) between Polar A300^™^ and SWA.

**Fig 4:**
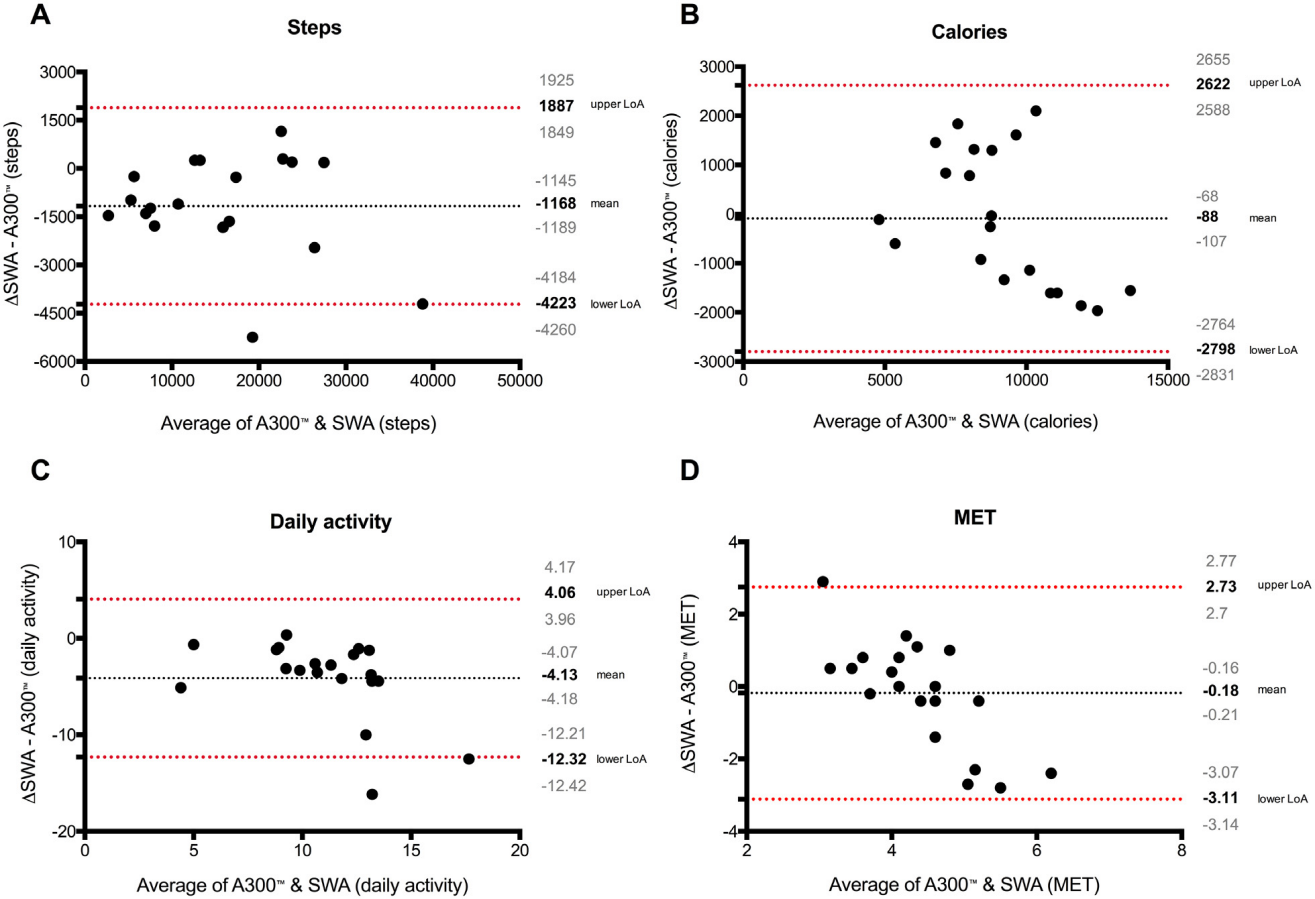
Bland and Altman plots with the mean over three days. Upper/lower limits of agreement and 95% CI (over/under the means) of steps (A), calories (B), daily activity (C) and MET (D) between Polar A300^™^ and SWA.

**Fig 5:**
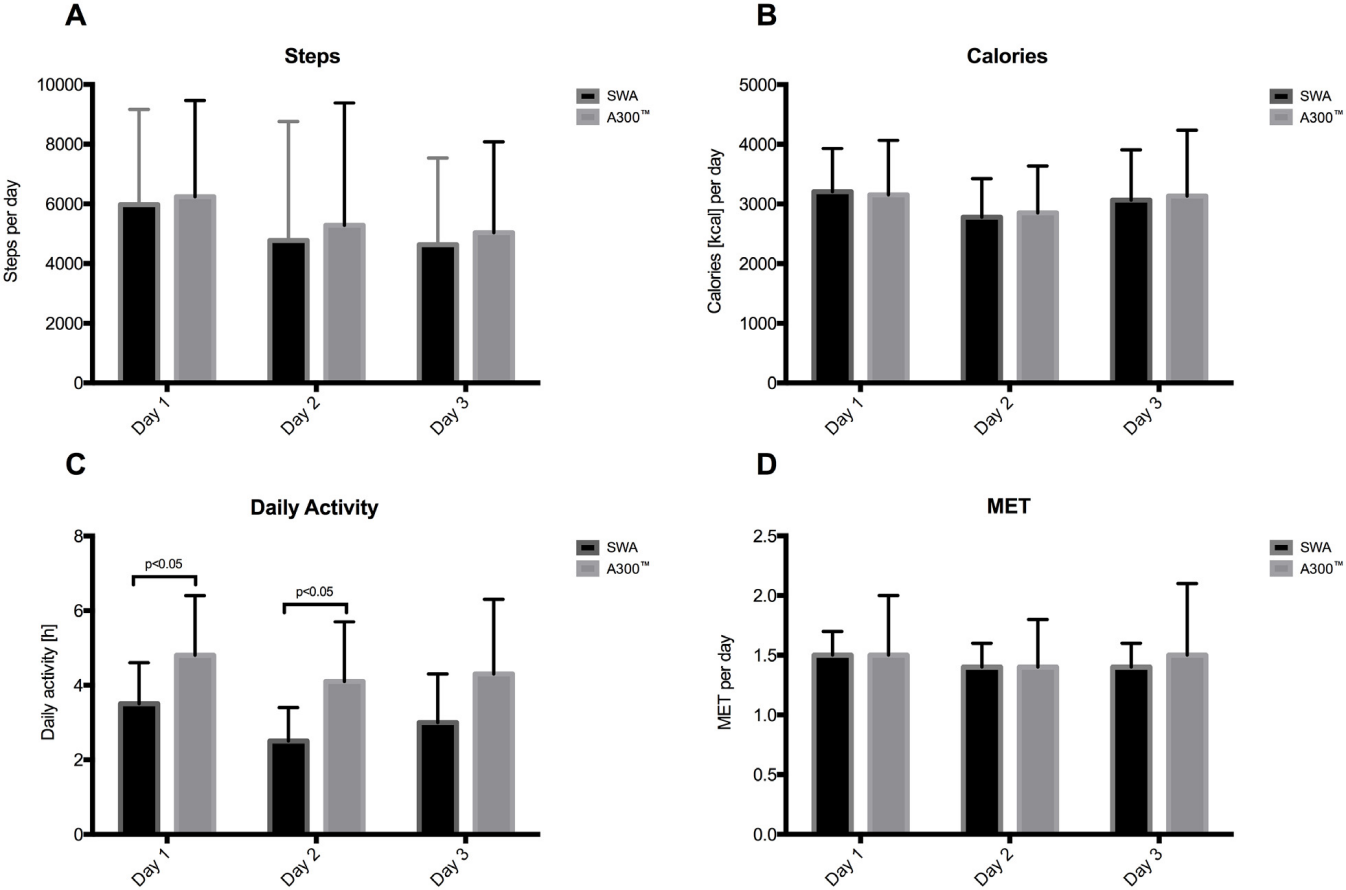
Day bay day comparison between Polar A300^™^ and SWA. Steps (A), calories (B), daily activity (C) and MET (D) (p<0,05).

Regression analyses of aggregated data over three days as assessed by both devices are shown in Fig 3. For comparison of both devices, Bland Altman plots were used and showed no systematic deviation. The data were largely within the limits of agreement (LoA) (Fig 4). A detailed day by day comparison including for steps, calories, daily activity and MET is given in Fig 5. A significant deviation can only be noticed in the daily activity time, whereas all other values show no difference. A day-by-day Bland Altman plot comparison of the data is given in S1 Fig. The 95% CI for mean difference of the A300^™^ compared with the SWA is in the range of 183 to 596 steps per day.

### Usability data

Table 3 shows the various domains for the usability and their results. Overall, in all 3 domains, significant differences between the devices were found in favor of the A300^™^ (p < 0.01).

**Table 3:** Domains and findings from the Post-Study System Usability Questionnaire (PSSUQ) (Scoring: 1 = strongly agree to; 2 = true; 3 = applies to part; 4 = neutral, 5 = does not apply to part; 6 = not applicable; 7 = strongly disagree).

Domain	Number of questions	System/Mean (Score 1–7)		p value
		A300^™^(Mean ± SD)	SWA (Mean ± SD)	
System Quality	6	1.46 ± 0.23	4.41 ± 1.18	< 0.01
Information Quality	6	2.41 ± 0.53	4.7 ± 0.7	< 0.01
Interface Quality	4	3.35 ± 0.62	5.33 ± 0.98	< 0.01

SD = Standard Deviation

## Discussion

The present study shows, that the low cost PAM A300^™^ had valid and usable properties to monitor physical activity in patients with COPD comparable to the well validated and frequently used SenseWear^™^. The latter served as a reference device. Our data confirms studies that compared other PAMs to the SWA. However, to our knowledge, this is the first study using the PAM A300^™^ [7]. Our results using measured steps, calories, and MET showed a high variance of differences between the two devices over 3 days. However, this was not significant. In the single day by day comparison we saw significant differences in the daily activity time, however by comparing the steps, calories and the MET we could not show a significant difference. The difference in the daily activity time may be influenced by different algorithm calculation of the producer [21] or systemic deviation witch largely remain in the range of +/- SD or the number of patients. Furthermore, we can present a high correlative relationship in steps, calories and MET in both devices. When taking the mean of 3 days’ data for comparison, our results were similar to other studies [4, 22]. It was shown that the 95% CI of the mean difference (steps/d) is less than 20% of the standard deviation of the control device (SWA) and thus fulfill the criteria of an equivalent system [23]. Physical activity plays a crucial role in the treatment of COPD [24], because a sedentary life style is correlated with increased mortality [25]. Therefore, it is helpful to give the patient access to affordable and precise PAMs that give reproducible and valid information about daily activity. However, many of these validated monitors have differently defined physical activity parameters. For example, the Actigraph^™^ shows the activity time and the so called VMUs (Vector Magnitude Units), the DynaPort^™^ indicates the exercise intensity and the time spent walking, standing, sitting, or lying, and the SAM^™^ shows the number of steps [26].

Using the questionnaire PSSUQ, we clearly demonstrated that the handling of the Polar device had advantages in system-, information-, and interface quality compared to the established Sensewear^™^ device. The COPD patients found the Polar device to be more intuitive and easy to handle providing comparable display information (clock, date, activity level). In particular, the wearing of the A300^™^ as a clock was perceived by the participants as pleasant. A high patient satisfaction ensures compliance with using the device thus allowing a long recording time [15]. Further studies for adherence to a PAM driven concept and the influence of the PAM A300^™^ in terms of activity increase compared to non-watch wearing groups still have to be done.

The present study has some limitations. It was an open-label, non-comparative study that included 20 patients/group. The number of included subjects, especially in the GOLD stages III and IV is small for the comparative purpose of the study. Since 100% of the included patients were above the crucial number of steps/day, we can provide solid activity tracker comparison data in all patients. Recently published data by Waschki et al. showed that COPD and chronic bronchitis patients had a significant reduction of daily activity over three years [25]. Since a reduction of activity occurs in all stages of COPD as well as in chronic bronchitis, the distribution of patients to COPD severity stages appears of minor relevance when comparing two activity trackers. A limitation of both devices is the generation of activity data by 3D-accelerometer sensors. It cannot be ruled out that slow device guided movements of the arm where the PAM is worn such as slow cycling, slow motorbike rides, slow car drives, and assisted wheel chair rides might be taken as active movements [27].

### Conclusion

The Polar A300^™^ is a mid-price activity monitor on the market, which can provide valid, precise, and reproducible estimates of physical activity in COPD patients compared to the frequently used reference device (SWA). Before recommending a general use of the A300^™^ further clinical trials in hospital and non-hospital settings are required. If elderly patients should be a target group for the PAM, feasibility studies in terms of self-analyzing of the data are mandatory. In terms of technical logging of data, we could demonstrate that the Polar data were comparable with the reference device Sensewear^™^. PAM might be considered as a long-term option in COPD, since this device may support an increase in physical activity levels by providing information and feedback on physical activity in patients with COPD. This was not addressed in the present study.

## Supporting Information

S1 Fig Day to day Bland Altman plot comparison between Polar A300^™^ and SWA. Steps (A), calories (B), daily activity (C) and MET (D) (p<0,05).
(TIFF)
