# Supplementary figures and images for: Validity and Usability of Physical Activity Monitoring in Patients with Chronic Obstructive Pulmonary Disease (COPD)
Source: PLoS One. 2016 Jun 15;11(6):e0157229. doi: 10.1371/journal.pone.0157229 (PMC4909270; doi:10.1371/journal.pone.0157229)

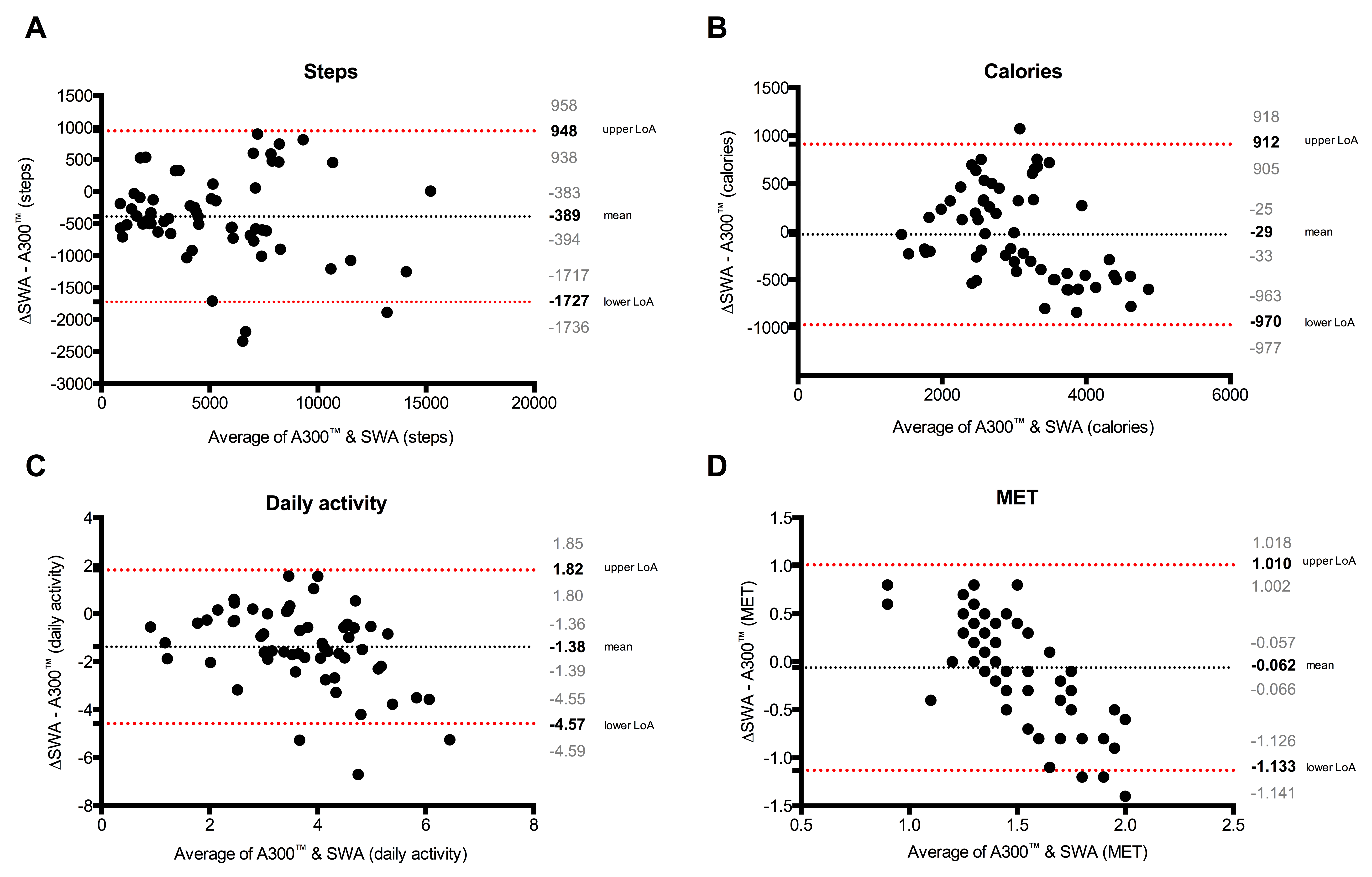

Supplement: S1 Fig — Steps (A), calories (B), daily activity (C) and MET (D) (p<0,05). (TIFF) [file pone.0157229.s001.tiff]
